# A Rare Case of Cutaneous Meningioma Arising on the Scalp: Diagnostic Challenges, Immunohistochemical, and Molecular Features

**DOI:** 10.7759/cureus.94117

**Published:** 2025-10-08

**Authors:** Kainat Memon, Richard J Digby, Azzam Ismail

**Affiliations:** 1 Department of Hematology and Oncology, Leeds Teaching Hospitals NHS Trust, Leeds, GBR; 2 Department of Histopathology, Leeds Teaching Hospitals NHS Trust, Leeds, GBR; 3 Faculty of Medicine and Health, University of Leeds, Leeds, GBR

**Keywords:** cutaneous meningioma, diagnosis, histopathology, immunohistochemistry, molecular features

## Abstract

The differential diagnosis for scalp lesions is broad, ranging from simple cysts to tumors. We present the case of a 49-year-old woman with a scalp lesion initially suspected to be an epidermoid cyst but later diagnosed as cutaneous meningioma on biopsy. As a rare form of extracranial meningioma, this case underscores the importance of correlating clinical findings with histopathology, such as integrating morphological features, immunohistochemical markers, and molecular studies, to establish a diagnosis in challenging cases with inconclusive clinical or radiological findings.

## Introduction

Meningioma is the most common adult CNS tumor, typically presenting as a slow-growing intracranial mass. These tumors usually arise in classic meningeal locations, which facilitates diagnosis. Rarely, meningiomas can occur extracranially, as in cases of cutaneous meningioma, an uncommon manifestation that poses diagnostic challenges. Cutaneous meningioma most often develops on the scalp [1] and typically presents as a solitary, firm, gray, or white subcutaneous nodule. Its broad clinical differential diagnosis includes nevus sebaceous, cyst, fibroma, glioma, lipoma, and alopecia areata. Because cutaneous meningiomas can closely mimic a variety of common skin lesions, clinicopathological correlation is essential for accurate diagnosis and appropriate management [2].

## Case presentation

A 49-year-old female presented to her general practitioner with a large, smooth, cyst-like swelling on the right side of her head that had been present for two years. The swelling had gradually increased in size and become more painful, though there were no signs of infection or inflammation. An excision biopsy was performed under local anesthesia with a clinical suspicion of an epidermoid cyst. During excision, an unencapsulated subcutaneous mass measuring 3 × 2 cm was identified, and the intraoperative impression was that of a lipoma.

**Figure 1:**
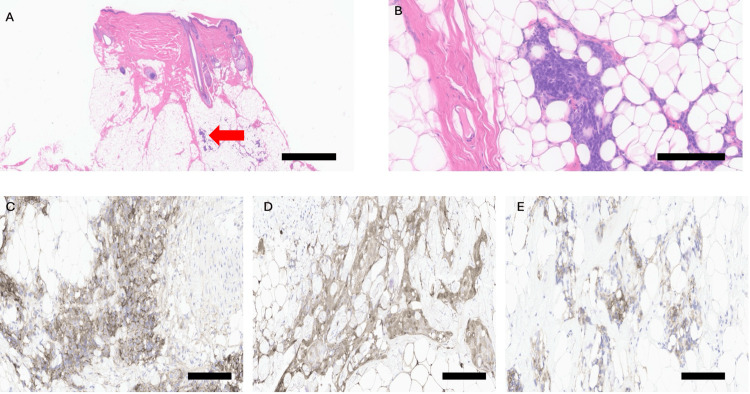
Histology and immunohistochemistry of the scalp lesion (A) Low-power view of the scalp section showing a nested proliferation of epithelioid cells in the subcutis (red arrow). Scale bar: 2 mm. (B) 20× view showing syncytial cells with round to oval nuclei and intranuclear pseudoinclusions, morphologically consistent with meningioma. Scale bar: 0.2 mm. (C) EMA staining showing foci of strong membranous positivity. Scale bar: 0.2 mm (D) S100 staining showing diffuse weak positivity. Scale bar: 0.2 mm. (E) CD56 staining showing scattered focal weak positivity. Scale bar: 0.2 mm. EMA, epithelial membrane antigen

H&E-stained sections revealed a lesion within the subcutaneous fat composed of ill-defined aggregates and cords of rounded cells with moderate cytoplasm (Figure 1A). The cells exhibited a syncytial appearance with round, uniform nuclei and minimal cytological atypia. Focal areas showed intranuclear pseudoinclusions, and no mitoses or necrosis were observed (Figure 1B). Immunohistochemistry demonstrated diffuse positivity for vimentin and patchy positivity for epithelial membrane antigen (EMA) (Figure 1C), S100 (Figure 1D), and CD56 (Figure 1E). Weak focal positivity for smooth muscle actin was also noted. At this stage, the differential diagnosis included sarcoma and meningioma.

Methylation analysis was performed using the Infinium Methylation EPIC array (Illumina Inc., San Diego, CA, USA), and data were processed via the Heidelberg Brain Tumor Classifier (molecularneuropathology.org). The analysis reported a calibration score of 0.91 for meningioma, with no match for sarcoma. The copy number variation summary showed a gain of chromosome 12 and a loss of chromosome 22. The most common genetic alteration in meningiomas is functional loss of *NF2*, found in approximately 40-60% of cases, usually due to loss of chromosome 22, inactivating point mutations, or gene fusions [3]. The *MGMT* promoter was unmethylated. An RNA fusion panel detected no fusions, ruling out sarcomas characterized by such alterations.

The morphology and immunohistochemical profile, together with the EPIC array and Brain Tumor Classifier results, supported a diagnosis of meningioma with no features of malignancy. The tumor extended to the specimen margins and was classified as a WHO grade I benign lesion, with a favorable prognosis if completely excised. Complete surgical excision is the mainstay of treatment for cutaneous meningioma. Preoperative imaging is essential to exclude deeper extension, such as intracranial involvement or neuroaxis connection, which may necessitate complex surgical planning [1].

An MRI of the head confirmed the absence of intracranial lesions, and CT imaging showed no evidence of bony sclerosis or lytic change. The patient had residual swelling at the excision site, showing slow growth without neurological symptoms. Complete excision of the lesion has been planned by the plastic surgery team.

## Discussion

Meningioma is the most common primary CNS tumor in adults. Most cases are benign WHO grade I tumors and are generally curable through surgical resection. However, higher-grade meningiomas are more aggressive and tend to recur despite multiple surgeries [3]. Extracranial meningiomas are uncommon; cutaneous meningiomas, such as in this case involving the scalp, arise from meningothelial cells ectopically located in the dermis or subcutis [4].

Cutaneous meningiomas are classified into three distinct types. Type I (primary cutaneous meningioma) is typically benign, present from birth, and most often found on the scalp, face, or paravertebral region of children and young adults. These lesions originate from arachnoid cell rests displaced during embryogenesis into the cutis or subcutis and generally have an excellent prognosis with a low recurrence rate [2]. Type II usually presents in adults as a de novo lesion, occurring around sensory organs of the head or along cranial and spinal nerve pathways as a cutaneous extension of an ectopic soft tissue meningioma. These are thought to arise from arachnoid cells displaced along nerve sheaths. Type III represents an extension into the skin from a CNS meningioma infiltrating through bone or a bony defect [2]. Prognosis depends on the lesion type, with Types II and III generally associated with less favorable outcomes, particularly when there is deeper tumor involvement or incomplete resection due to organ compression or infiltration [2,4].

Extracranial meningiomas are rare, comprising less than 2% of all meningiomas, and may occur in the head and neck region, skin, lungs, or mediastinum [5]. Cutaneous meningiomas pose a diagnostic challenge due to their rarity, with most cases documented only as isolated reports [6]. Literature review reveals few reported cases, underscoring the importance of considering cutaneous meningioma in the differential diagnosis of scalp lesions, the most common site of occurrence. The clinical differential diagnosis is broad and includes epidermoid cysts, dermoid cysts, lipomas, pilar cysts, neuroectodermal tumors, and metastatic lesions [1,6,7]. Clinical assessment and imaging are often inconclusive, making histopathological and cytological evaluation essential for definitive diagnosis. The most common histopathological type, the meningothelial variant, is characterized by lobules, nests, and sheets of oval or polygonal meningothelial cells arranged in a whorled pattern [7].

In this case, the tumor was morphologically difficult to classify; thus, immunohistochemistry was crucial. Careful microscopic examination combined with immunohistochemical positivity for EMA and vimentin strongly supports the diagnosis of meningioma. Conversely, negative staining for cytokeratin, Melan-A, CD31, CD34, CD68, and desmin helps exclude epithelial, melanocytic, vascular, histiocytic, and myogenic tumors [1,7]. Similar to our findings, other case reports have emphasized the diagnostic value of EMA and vimentin positivity, together with morphology and clinical correlation, in confirming cutaneous meningioma [6,7]. However, our case was notable for its atypical findings, including positivity for S100 and smooth muscle actin, which required further investigation and integration of all results to reach a conclusive diagnosis.

While most meningiomas can be readily diagnosed using clinical, radiological, and histopathological criteria, atypical presentations, especially those occurring at unusual sites such as the skin, may necessitate additional immunohistochemical testing and methylation profiling to establish a definitive diagnosis.

## Conclusions

This case underscores the importance of considering rare entities such as cutaneous meningioma in the differential diagnosis of scalp lesions, as atypical presentations can result in delayed or incorrect diagnosis. It demonstrates the value of an integrated diagnostic approach that combines histomorphology, immunohistochemistry, and molecular analysis. Additionally, it highlights the potential role of methylation classifiers in the diagnosis of soft tissue tumors, particularly in cases where uncertainty remains after extensive immunohistochemical evaluation. Further research is warranted to validate the diagnostic utility of methylation-based tools such as the EPIC array for identifying cutaneous meningioma.

## References

[1] Miedema JR, Zedek D (2012). Cutaneous meningioma. Arch Pathol Lab Med.

[2] Lopez DA, Silvers DN, Helwig EB (1974). Cutaneous meningiomas—a clinicopathologic study. Cancer.

[3] Szulzewsky F, Thirimanne HN, Holland EC (2024). Meningioma: current updates on genetics, classification, and mouse modeling. Ups J Med Sci.

[4] Ragoowansi R, Thomas V, Powell BW (1998). Cutaneous meningioma of the scalp: a case report and review of literature. Br J Plast Surg.

[5] Jaiswal P, Jaiswal S, Chakrabarti S, Mukherjee A (2018). Primary type I cutaneous meningioma of the scalp: cytohistological and immunohistochemical features of a rare neoplasm. Asian J Neurosurg.

[6] Ramos L, Coutinho I, Cardoso JC, Garcia H, Cordeiro MR (2015). Frontal cutaneous meningioma - case report. An Bras Dermatol.

[7] Kishore M, Kaushal M, Bhardwaj M, Sharma N (2017). Cutaneous meningioma: a cytomorphological diagnosis. Indian Dermatol Online J.

